# Creating Peru’s patient zero: pandemic narratives through traditional and social media

**DOI:** 10.1590/S0104-59702023000100049

**Published:** 2023-09-18

**Authors:** Alejandra Ruiz-León

**Affiliations:** i Georgia Institute of Technology Atlanta GA USA Arl@gatech.edu PhD Candidate, Georgia Institute of Technology. Atlanta – GA – USA Arl@gatech.edu

**Keywords:** Covid-19, Peru, Patient zero, Social media, History of medicine, Covid-19, Perú, Paciente cero, Medios de comunicación sociales, Historia de la medicina

## Abstract

During the covid-19 pandemic, authorities, journalists, and the public used the term patient zero to refer to the first diagnosed patient. However, experts describe the term as imprecise because it equates the first infected patient with the first identified one. Although the term’s inaccuracy, patients zero became relevant actors and sources of information during the pandemic. This was the case with the Peruvian patient zero, who had public media participation and opened his Instagram to establish a communication channel with the public. Despite knowing the term’s inaccuracy, he felt responsible for the audience and sought to give his testimony. The Peruvian case shows how patients zero respond to the public interest and establish their agency through traditional and social media.

## Resumen

El coronavirus hizo que autoridades, periodistas y público designaran “paciente cero” al primer diagnosticado, aunque los especialistas calificaran al término como impreciso por equiparar el primer paciente infectado con el primero identificado. A pesar de esa inexactitud, pacientes cero se tornaron actores y fuentes de información relevante durante la pandemia. Fue el caso del paciente cero peruano, que participó en los medios de comunicación y abrió su Instagram para establecer un canal con el público. Conociendo la inexactitud del término, asimismo trató de dar su testimonio para aclarar la audiencia. El caso peruano muestra cómo pacientes cero responden al interés público y establecen sus acciones mediante los medios tradicionales y sociales.

On May 31, 2021, Peruvian President Francisco Sagasti announced the results of an independent study conducted by Peruvian scientists and health authorities that revealed the confirmed number of covid-19 deaths in the country since the beginning of the pandemic (Chávez, 31 May 2021). The new death toll shown in the study placed Peru at the top of the list of the most affected countries per capita, with the loss of more than 184,000 Peruvians. The report became news, both internationally and domestically. For Peruvians, it confirmed the widespread suspicion that the actual number of deaths exceeded the official data provided by the government (Perú es…, 1 June 2021). For international observers, the report prompted questions as to whether their reports and official data accurately captured the impact of the pandemic.

New information, such as the updated official death toll in Peru, calls for an analysis of the successes and failures in responses to the pandemic, which does not yet have an end date. As Marco Cueto (2022) reflects on *Salud en emergencia,* historical research of the events that happened during the covid-19 pandemic allows us to analyze the social process that shaped the pandemic, such as the establishment of insufficient policies or how the authorities feed the audiences with false promises or incomplete information. One area of interest in the history of medicine is how the first days of the pandemic developed, which scholars see as critical to understanding the pandemic’s progression. This paper focuses on the patient zero figure during the early days of covid-19 pandemic in Peru and how media and authorities used the term to identify the country’s first person who tested positive for coronavirus.

The pandemic in Peru technically started on the morning of March 6, 2020, when President Martín Vizcarra announced during a televised media conference that “the first case of coronavirus infection has been confirmed” (Presidente…, 6 mar. 2020). Other countries in the region, such as Chile and Argentina, had already reported coronavirus cases, making the identification of the first Peruvian patient only a matter of time and an expected sign to declare the onset of the pandemic. During the first days, the term patient zero was broadly used by politicians, journalists, experts, and even the first patient of covid-19, to refer to the first coronavirus case in the country. TV Perú, Peruvian public television, used the term patient zero from the first reports of the patient, mentioning that “patient zero revealed that he sought dismissal in the clinic up to three times” (Covid-19…, 9 Mar. 2020). Peruvian television announced the patient’s first interviews as “the testimony of the patient zero” (Día D, 9 Mar. 2020), and authorities such as the minister of Health used the term when announcing the patient’s discharge (Ministra…, 16 Mar. 2020).

The Peruvian case shows how covid-19 patients zero openly told their stories as the first persons identified as being infected with the novel coronavirus. Calling the first patient by the term “patient zero” preserved the person’s identity while informing the population of the start of the pandemic. Legal and moral practices prevented authorities and the media from revealing personal information that could identify the patients. As this paper will address, however, patients zero did not always remain anonymous and, in some cases, they participated in interviews, public health campaigns or even gained momentary fame.

The use of the term patient zero in disease outbreaks continues, despite medical experts stressing its lack of scientific accuracy and negative implications (Giesecke, 2014; McKay, 1 Apr. 2020). Historians of medicine have studied how societal aspects shape medical events, stressing that social interpretations influence our understanding of a disease beyond scientific information and medical expertise. This paper builds on previous research on patients zero in diseases like aids, flu, and Ebola, among others (Coltart et al., 2017; Marineli et al., 2013; McKay, 2017). When looking back to the actions that shaped the pandemic, historians should look for new ways patients establish their agency. Historians have previously used patients’ diaries to understand their perspectives, even if personal diaries can be considered subjective (Condrau, 2007). Scholars should now look into social media as a platform that patients use to tell their side of the story and to control their narrative. Unlike traditional media, social media offers patients direct communication with the general public and provides a more direct connection with both positive and negative outcomes.

This research explores how social media has given patients zero new means for establishing their agency in situations where they have been under intense public scrutiny. Disciplines such as science communication, history of medicine, and ethics have studied the use of social media for patient advocacy (Househ, Borycki, Kushniruk, 2014). Social and traditional media became outlets for patients “to comfort the public with the hope that the virus would be controlled” (Pascual Soler, 2021, p.71). In the case of the Peruvian coronavirus patient zero, social media served as the primary platform to share information that was unique to that person but comforted the public when scientific certainty was scarce. However, for several reasons, social media has not always been the primary communication for patients zero in previous outbreaks. For example, in the case of aids, the patient zero did not participate in interviews as the media only knew his name after his death . In the case of the 2014-2016 Ebola outbreak in West Africa, the patient was a minor and, in earlier times, social media did not exist, as in the case of typhoid fever.

The concept of patient zero is used because it holds enough information for the audience to understand the relevance of this person’s testimony and potentially respond to questions that experts cannot answer. For example, the Peruvian patient zero explains how he knew he was possibly not the first Peruvian with coronavirus but rather the first one to be identified (Día D, 12 Apr. 2020). If he was not the first patient, there were already other patients, which indicates that the government’s actions were insufficient to contain the virus entry. The scientific research later confirmed the denominated patient zero’s suspicion that he was not the first person in Peru to be infected with the virus. Phylogenetic and epidemiological research made with samples from the first months of the pandemic shows multiple virus entries into Peru, confirming that not all cases came from patient zero (Juscamayta-López et al., 2021; Padilla-Rojas et al., 6 Sep. 2020). Authorities and experts discussed this scenario; however, the ideas of the initial date of the pandemic and the existence of one introductory case prevailed.

The idea of a patient zero still holds meaning for society, making it appealing to provide information when scientific research cannot provide facts. The new ways that society has used the concept of patient zero during the covid-19 pandemic make this a relevant topic for historians of medicine and public health officials who need to assess the dimension of risk in using this term for pandemic communication. Focusing on the Peruvian case, we see how social narratives about the pandemic circulated on both traditional and social media. The latter offered patients zero new platforms to advocate for themselves and for audiences to acquire medical information beyond those provided by authorities and experts.

This research builds on different sources to understand the role of patients zero during the onset of the pandemic and how they established their agency after becoming a subject of interest. To do this, the essay draws on academic literature dealing with the social construction of the term patient zero and its role in previous outbreaks, as well as the work of historians of science on patient zero. The analysis includes Peruvian and international media, examining how newspapers and online information reflected the identification of the first case of coronavirus in different countries. Additionally, the essay analyzes the public communication of the “Peruvian patient zero,” including media appearances and social media publications, to show how narratives of heroism and national strength prevailed in journalists’ and authorities’ statements and how the patient’s agency was established through traditional and social media. Overall, the research analyzes the use of the term patient zero during the beginning of the covid-19 pandemic in Peru. It also shows how the image of the first patient was shaped by governmental authority’s statements, media coverage, and the patient’s agency established through traditional and social media.

## The concept of patient zero

When a part of the general public uses a technical term, they may give it a new meaning, different from what experts use within their communities. Medical and scientific groups use technical terms to share information within their professional environments, and these terms relay specific information. However, expert communities cannot prevent technical jargon from being used in other ways by non-experts. The general public can use and adapt these terms. During the pandemic, technical words such as quarantine, inoculation, or contact tracing ceased to be used exclusively by professional groups as society included them as part of their daily language.

Communities also adopt new terms to describe medical situations, even when these terms do not have scientific meanings or medical experts do not use them. This was the case during the covid-19 pandemic with the term patient zero, which authorities and media used, while scientists avoided it because it is inaccurate. Experts stressed that during previous outbreaks, the term patient zero created adverse outcomes such as associating a disease with moral responsibilities or with minority groups (McKay, 1 Apr. 2020). During the covid-19 pandemic, journalists and laypeople continued to use the term because it held meaning for society and announced the onset of the pandemic. Understanding the term’s history and how it serves society is essential since the media and the public will likely use it in future disease outbreaks.

The term patient zero refers to the first person identified as a patient with a disease. The historian of medicine Richard McKay explains how the term is “often used interchangeably for three different scenarios: the first case noticed, the first case here, and the first case ever”⁠ (McKay, 1 Apr. 2020). For the scope of this paper, we will focus on the term’s first two interpretations. In Peru, media and authorities used the term patient zero to refer to the first person infected with the virus and the first Peruvian case. The two interpretations differ from “the first case ever,” meaning the first case in Wuhan, China, referring to the virus’s origin.

Epidemiologists discouraged media outlets from using the term patient zero because of its inaccuracy. Besides the different meanings of the term, another limitation is that the term does not differentiate between the first infected patient, the first symptomatic patient, and the first person identified as a patient. Moreover, the term gives the illusion that it is always possible to identify the first infected person when there might not be enough evidence to confirm the original patient contracting a disease. Instead, epidemiologists advocate for the terms “index case” and “primary case.” According to *A Dictionary of Epidemiology*, an index case refers to “the first case in a family or other defined group to come to the attention of the investigator” (Porta, 2016a, p.146). The primary case refers to “the individual who introduces the disease into the family or group under study. Not necessarily the first diagnosed case in a family or group” (Porta, 2016b, p.225).

The term patient zero is less precise than the terms index case and primary case because it ignores the possibility that the patient that introduced a disease into a group might not be the one identified by authorities. This is one reason the term patient zero is disadvantageous for technical communication. For the community, it provides a sense of security about the starting date of the pandemic and the efficiency of case surveillance, even when this information is undetermined. The terms index case and primary case acknowledge that the first captured patient might not be the first infected patient, which is a probable scenario in an outbreak. The correct term to describe the Peruvian patient zero should have been index case because investigators identified him as the first.

The covid-19 particularities make the term patient zero more complex because patients could be symptomatic or asymptomatic. When using the term patient zero, there is an interest in differentiating between sick and healthy people, referred to as the process of becoming a patient (Porter, 1985). Covid-19 asymptomatic patients do not show symptoms and are only considered sick with a positive test. Moreover, the notion of asymptomatic patients was uncommon at the pandemic’s beginnings (Schuetz et al., 17 Dec. 2020). Hospitals tested patients only after they developed symptoms or revealed a history of travel to a country with communal virus transmission (WHO, 2020). The patients zero who sought diagnosis had to contact the authorities because the progression of the disease was unknown, and the focus was on tracing the patient’s close contacts.

The history of the term patient zero helps us understand the flaws of this concept and why its use during the pandemic was problematic. In the book *Patient zero and the making of the AIDS epidemic*, McKay (2017, p.28) details the creation of the concept in 1980, when the Centers for Disease Control and Prevention (CDC) monitored the first cases of aids in the United States. The CDC used the label “Patient O,” where the O referred to a patient “Out of California,” with no zero, as it would later be known. The term patient zero was not born to signal the first patient but rather the location of one patient from a cluster of cases in California. McKay explains how the media used the concept to create curiosity, not for scientific objectives. The journalist Randy Shilts was one of those responsible for amplifying this concept by promoting the image of hunting for the person that supposedly brought the virus to the United States. Shilts embarked on a professional and personal search, revealing the identity of Gaëtan Dugas, who became wrongly known as the patient zero in the North American aids epidemic (McKay, 2017). The term gained additional connotations through the years; as McKay (1 Apr. 2020) expressed, the term denotes a sense of urgency and resembles military terms such as “hour zero” or “ground zero.”

Society gives meanings to specific terms, such as patient zero, in complex situations like epidemics. Paula Treichler coined the term “epidemics of significations” to illustrate how in medical situations, society awards meanings to terms that influence societal understanding and approaches to medical conditions (McKay, 2017, p.5; Treichler, 1987). These concepts result from co-production, where scientists, doctors, journalists, and audiences create and use new words with purposes beyond the expert’s use. For example, the term patient zero originated to designate the location of a group of patients. However, it changed its meaning when the “o” was misinterpreted for “0,” which led to the interest in identifying aids’s primary case. Then the social meaning of the concept became more representative than its historical meaning.

Although the term patient zero started during the aids epidemic in North America, this was not the first time scientists and media focused on the first known cases of a disease. The most well-known patient zero case, or correctly, an index case, was Mary Mallon, known as Typhoid Mary, whom doctors identified as a super spreader of typhoid fever. In Mallon’s case, the term healthy carrier added a medical component and a moral one. She was stigmatized and portrayed as irresponsible for making others sick, as well as for being a woman immigrant who could not understand the scientific rationale behind the measures taken to seclude her from society (Marineli et al., 2013). There has also been an interest in identifying the patient zero in more recent epidemics, as is the case of the Ebola outbreak in 2014-2016, where scientists used the patient’s DNA sequences to trace the primary case, Emile Ouamouno, a two-year-old from Guinea (Coltart et al., 2017).

The media and communications from experts also affect how the public perceives patients zero. In Peru, doctors described the first aids patients as irresponsible, shaping the social understanding of the disease and impacting the access to diagnosis for fear of being identified (Lan Ninamango, 2021). Similarly, during the cholera epidemic of the 1990s, public health interventions focused on personal responsibility instead of the lack of infrastructure that promoted the spread of the disease (Cueto, 1997). In these cases, the public saw the patients’ responsibility as crucial for their survival, and doctors and authorities supported and spread these narratives.

Even when the term patient zero gave a sense of security and control, many historians and public health experts indicated the risks of misusing this term, calling it toxic and inappropriate, based on the stigma and shame experienced by patients in previous pandemics (McKay, 1 Apr. 2020). However, this did not limit the use of the term during the covid-19 pandemic. Moreover, people might continue to use the term in future epidemics. Understanding the past and future use of the term patient zero is essential, particularly in how these patients establish their agency when interacting with broader audiences.

## Coronavirus patients zero in the news

During the last weeks of 2019, news outlets worldwide began to cover the appearance of a new respiratory virus in China. The virus became a global event when virtually every country detected coronavirus patients inside their territories. Authorities and media channels had the opportunity to prepare for the virus’s arrival by coordinating public health strategies and communicating prevention narratives. During the first months of 2020, countries such as Peru took measures to delay the virus’s circulation and contain the first cases to avoid the communal spread of the virus (Perú, Jan. 2020). Many countries followed a zero-case approach at the beginning of the pandemic to limit the spread and reach zero cases, a strategy that only a handful of countries continued in 2022 (Marshall, 3 May 2022).

There was a growing interest in identifying the patient zero from each location or country as the coronavirus spread worldwide. The coverage of patient zero in different countries shared a common interest in showing the patient’s experiences. The risk of these approaches was prioritizing the patient’s capacity to follow the rules instead of the lack of medical infrastructure that influenced the patient’s outcomes. The interviews with patients zero responded to social narratives of national pride, medical advantages, and other ideas promoted by governments and authorities. A common discourse was to interpret the pandemic as a war against an invisible enemy, where the patients zero became part of the heroes that helped us survive the pandemic with their work and example (Pascual Soler, 2021).

In the case of the first patient ever, we see how the lack of a person identified as an index patient fueled critics of the Chinese government and led to disinformation, misinformation, and racist claims toward Asians (Wang, Santos, 2022). The lack of information regarding China’s patient zero contributed to the aura of conspiracy and uncertainty regarding the pandemic’s origins (Worobey, 3 Dec. 2021). While the first coronavirus patient ever remained unknown, other countries rushed to identify their first patients.

Two cases of patients zero with several media appearances were the denominated Italian and New York patients zero. The first identified coronavirus case in Italy was Mattia Maestri, a 38 years-old man from the country’s northern region. Media reports described him as a middle-class working person, representative of Italians, who had not traveled abroad but was infected and spent some time in hospital. He described himself as a messenger of positivism and social awareness with a moral duty to his community. He later became a symbol of national resilience, participating in public health campaigns, raising funds for patients and being described as exemplary (Politi, 16 Sep. 2022). In New York, the media coverage of patient zero replicated similar narratives of heroism and personal responsibilities. The patient zero from New York gave an interview accompanied by his family. The media described him as a working person, dedicated to his family, who lived in the New York suburbs, and as someone who had no contact with foreign visitors. In the interview, the media showed his family bonds as crucial for his recovery (Brody, 5 Mar. 2021). Like the Italian patient zero, the press portrayed the New York patient as an exemplary citizen concerned for others’ health and committed to controlling the pandemic.

However, not all media portrayals of patients zero were positive. One of the most notable case was a Vietnamese socialite, referred to as fashion’s patient zero (Friedman, 11 Mar. 2020). In March 2020, she participated in European fashion shows before knowing she was infected. The audience saw her as irresponsible for spreading the virus, and she received attacks on her social media, forcing her to close her channel (#BAZAARTalks, 31 May 2020). These attacks referred to her wealthy status and Asian identity, which were part of a larger trend of racism towards Asians during the pandemic.

In Peru, the media also reported xenophobic attacks against Asian communities. Ragas and Palma note how these communities were not subject to violent attacks in Peru, as was seen in the United States. In Peru, xenophobic attacks were limited to messages replicated by small radical groups rather than the general population or authorities. According to Ragas and Palma (2022), the collaboration of the Chinese government in controlling the pandemic was seen in Peru as positive and may have helped to limit the attacks on Asian communities.

Social aspects influenced how the media portrayed patient zero. Gender, age, ethnicity, and social status of the patients were frequently included in their media participation and influenced how audiences perceived patients zero. McKay explains that minorities are at risk of being portrayed as irresponsible because they are “judged to have disobeyed community standards” (McKay, 1 Apr. 2020). Even when the identity of patients zero was unknown, as in China, there was still an interest in identifying them to gain more knowledge on how the pandemic began (Calisher et al., 7 Mar. 2020).

Peruvian media covered the first covid-19 cases outside China with a sense of urgency, reporting on how health agencies in Europe and South America had identified positive cases and how Peru was preparing for the virus’s arrival (El Coronavirus…, 4 Mar. 2020). Time was running out before confirming the first case in Peru, and the hunt for patient zero had begun. National newspapers such as *La Republica* featured headlines such as “Coronavirus every time closer to Peru” (El Coronavirus..., 4 Mar. 2020). The government also made several media appearances, with the minister of Health announcing the government’s covid-19 containment plan (Elizabeth…, 3 Mar. 2020).

## Finding the Peruvian patient zero

The news from international patients zero created an environment of expectation in Peru, where people saw the arrival of the coronavirus as inevitable. The government responded with the publication of its covid-19 protocols, and the media followed with information about the virus. The public expected the announcement of the first patient; however, nobody could predict the virus’s impact in the following months. The public interest in patient zero focused on his/her correct detection and the development of his/her infection since the public would measure the virus’s severity based on this, even when each person responds differently to the virus.

Identifying the first covid-19 patient in Peru changed the government’s response to the virus. As the historian Jorge Lossio details, the government’s first strategy was to prevent the entrance of the virus. To pursue this goal, they purchased covid-19 tests and screened passengers traveling to Peru for coronavirus symptoms, such as fever and coughing. After the first case, the government’s response redirected to “prevent exponential spread, inform the population about the coronavirus and improve hospital infrastructure” (Lossio, 2021, p.582). In the following days, the president announced strict measures such as closing schools, suspending flights, and a national lockdown that extended over a period of a hundred days.

The government’s rapid response and advancements in health infrastructure initially gave the public hope that the country could control the pandemic. Before the pandemic, experts recognized Peru for its economic growth and progress on global development goals, such as universal health coverage. These improvements were insufficient to overcome the institutional fragmentation of the Peruvian health system, and disparities in access to healthcare became more pronounced during the pandemic (Gianella et al., 2020). As the pandemic progressed, the healthcare system collapsed, making it impossible to provide adequate treatment for all patients, resulting in a high number of deaths (Gianella, Gideon, Romero, 3 Apr. 2021).

The first news of potential cases was in January 2020, when Peruvian media reported two suspicious cases of coronavirus involving Chinese visitors (Ministra…, 27 Ene. 2020). Only a few countries had identified coronavirus cases at that time, which limited the information on procedures and testing methods. First, three visitors from Wuhan and their Peruvian translator presented symptoms associated with the coronavirus, but public health officials and doctors reported that they did not have covid-19. The news did not specify if they tested explicitly for the novel coronavirus. Two other Chinese tourists presented symptoms while visiting Cusco, but they did not come from Wuhan, which was one of the required characteristics to signal someone as a covid-19 patient at the time. The news mentioned a negative laboratory test but did not specify if sensitive coronavirus tests were then available⁠ (Cusco..., 30 Ene. 2020). More than two years after doctors identified these potential index cases, it is impossible to confirm whether these visitors were positive for the coronavirus. However, this shows how limited the detection protocols were in January 2020. During that time, Latin American authorities and the public did not see the virus as an immediate threat. They saw news from China as distant, which was reflected in the lack of follow-up in media coverage of these patients. However, from January to March, when the president announced the first confirmed Peruvian covid-19 case, the situation, and public perception of it, had changed dramatically since neighboring countries had already identified covid-19 patients and because cases were growing worldwide.

Peruvians woke up on March 6, 2020, to a presidential emergency message. Martin Vizcarra, president of Peru, announced that the National Institute of Health (Instituto Nacional de Salud, INS) identified the first coronavirus patient in Peru, and health authorities followed the mandated protocols. Vizcarra described patient zero as “a young man of 25 years old that had a history of travel to Europe” (Presidente…, 6 Mar. 2020). The president did not disclose his name or any identifiable information because legal regulations prevented it. In a later interview, the patient zero said that the president’s message aired before the health authorities confirmed that he was positive for the coronavirus. The details shared by the president were enough for patient zero to recognize himself as the first case (Día D, 12 Apr. 2020).

The public identification of the Peruvian patient zero sparked interest from more than just epidemiologists. The general public and authorities wanted to know patient zero’s traveling schedule, and close contacts, among other information that might offer a greater understanding of the virus. As a result, many of his personal details were shared even though they did not relate to the coronavirus. For example, without sharing his name, the minister of Health disclosed that he was a commercial pilot and had been in Europe for his vacations, not for work. This is irrelevant, since the virus could have infected him regardless of the motive of his travel. Latam Airlines, the Peruvian patient zero’s employer, responded by clarifying that he did not travel as part of his work activities (Latam…, 6 mar. 2020). With this statement, the company sought to separate itself from the patient zero and assure passengers that the infection had not occurred on their planes. Yet, Latam could neither confirm nor deny that this was the case.

The Peruvian patient zero gave his first anonymous interview on the Sunday primetime news show *Día D* conducted by the journalist Pamela Vértiz on March 8, 2020. During the interview, the journalist called him “Pedro,” a fake name to preserve his privacy. The journalist assured the audience that she was interviewing him with “the needed reservation” (Día D, 9 Mar. 2020), appealing to the potential moral and legal consequences of exposing a patient’s name on national television. During the interview, “Pedro” was referred to as the covid-19 patient zero of Peru, and he explained how he became the first detected patient. The patient described visiting several European countries and having symptoms after arriving in Peru. Given these circumstances, he could not conclude if he got sick abroad or once he arrived. The journalist explained that the patient visited a private clinic three times, but on every occasion he was incorrectly diagnosed with a common cold. “Pedro” stressed that he received a correct diagnosis only after he contacted the INS. The INS sent a team to his house to collect a sample, and after his positive diagnosis, they provided him with medical guidance. The patient refused to name the clinic and doctors that misdiagnosed him; moreover, he aimed to inform the population that they had to be persistent if they suspected a covid-19 infection. The journalist emphasized this idea, which presented patient zero as a responsible and concerned citizen who went above and beyond to get the correct diagnosis when others might not have done so.

During the March 8 interview, the journalist Pamela Vértiz did not mention that she knew the patient zero personally. She revealed this information during a later non-anonymous interview with the patient on April 12 (Día D, 12 Apr. 2020). Their relationship explains how the journalist could get an exclusive interview with the patient when the authorities had not disclosed his identity to the media. In both interviews, the media portrayed him as a responsible person who had reached out to the authorities even when doctors dismissed him. The fact that she knew him personally might have affected how the narrative about him was constructed and, by extension, how the public perceived him.

Although the first interview with the patient zero portrayed him as concerned about the spread of the virus, many members of the public rejected this portrayal. They criticized him even without knowing his identity. *Día D* reposted the interview of March 8 on their social media, where people reacted to his testimony. Some perceived him as responsible for reaching out to authorities and following instructions. Nevertheless, many viewed him as irresponsible for traveling to Europe in the first place, when the virus was known to be circulating there. A comment on *Día D*’s Facebook responds to the interview’s framing of the patient zero as a hero: “It is not an act of heroism to tell that he irresponsibly traveled to Peru from places where he had been exposed to the virus” (Neyra Schenone, 9 Mar. 2020). Other users defended him from such criticisms saying, “The young man is helping by providing information about the virus at the risk of revealing his identity” (Sherly, 10 Mar. 2020).

The public viewed patient zero’s socioeconomic status as relevant because it offered information on how he got infected and later recovered. Despite the anonymity of the patient’s first interview, the audience recognized him as young and upper class. A comment read, “the coronavirus came to our country because of the people with money, because the poor do not travel anywhere … rich people are to blame” (Arcentales, 10 Mar. 2020). After his negative test, the authorities showed him as an example of how patients would experience and recover from the coronavirus. The minister of Health called him a model patient who showed that “most cases will pass as a mild respiratory infection” (Ministra…, 16 Mar. 2020). The patient zero’s experience was not representative of the experiences of many coronavirus patients. He had access to medical support, the health authorities closely monitored him (Jochamowitz, León, 2021), and he had the financial means to stay home while recovering.

Social media was a space for the audience’s comments and reactions, and where narratives about the pandemic were built and shared. While individual reactions to the pandemic may be limited to personal networks, social media can amplify opinions to larger audiences. On these platforms, people engaged in conversations about the pandemic’s impacts and were exposed to narratives that traditional media may not have covered. For example, while traditional media may not have commented on the socioeconomic status of Peru’s patient zero, this topic was a recurrent subject of discussion on social media as people interpreted it as a factor in the patient’s infection and recovery.

The Peruvian patient zero went beyond giving anonymous interviews with the media to share his testimony. He also established a direct communication channel with the audience by opening his Instagram account to give a public statement and updates on his health status after testing negative for the coronavirus multiple times. In his first publication, he used Instagram Stories, which disappeared after twenty-four hours but were later saved in his main profile (Zevallos, s.d.). Because Instagram Stories do not support the direct sharing of publications, the public had to take screenshots and tag the patient zero’s account to quote him. The audience could message him directly even though Instagram Stories do not support public comments. Since he used his personal account, the public saw his previous publications, which included photos of his family and friends. The publications about the coronavirus quickly became viral, and many Instagram users shared them, which put attention on his profile.

The patient zero reinforced his status as a person of interest as he shared his personal experience of being the first identified coronavirus patient in Peru on his Instagram account. He self-identified as the patient zero, acknowledging the significance of this term for both him and the public, who quickly recognized the value of his testimony. His publication titled “*quédate en casa*” (stay at home, in English) was the Peruvian government’s slogan for the coronavirus campaign. The publications started with “Hi, I am Luis Felipe, the case 0 of coronavirus in Peru.” They included detailed information about his trip to Europe, his symptoms, details of his infected relatives, the medicines that doctors prescribed, and the names of the doctors who treated him, among other information. He said people should not use his publication to self-medicate since he was not prescribing these medicines, just sharing his experience. He also stressed the importance of isolation to limit the spread of the virus and follow the government’s protocol. Finally, he posted a negative result of the molecular coronavirus test and concluded that he had beaten covid-19.

The public’s interest in patient zero was extensive and long-lived, continuing throughout the pandemic and impacting those around him. The public was also interested in his family and the doctors who treated him while he was sick. In later posts, he included a video of his seven-year-old nephew, who had previously tested positive for coronavirus, asking people to stay home. The patient zero also posted about his grandparents and how they survived the coronavirus despite being at higher risk because of their age. Although the publications had more than two thousand likes, the public could not comment on the Instagram posts because the comments were disabled, a common strategy when social media users expect an adverse reaction. On his social media, the patient zero introduced doctor Ramos, an infectious diseases physician from the Ministry of Health who was part of the covid-19 team. Doctor Ramos used his social media to share information about the virus while collaborating with traditional media (Ramos Correa, s.d.). In 2021, doctor Ramos died during the second wave of the coronavirus in Peru. The media reported his death as the “doctor who treated covid-19 ‘patient zero’” (Redacción EC, 17 Mar. 2021), demonstrating that more than a year after the first coronavirus case in Peru, the news recognized the doctor by his relationship with patient zero.

Patient zero received many negative comments on social media, but he considered them part of his responsibility to help inform the public. It is undetermined how many people saw his posts and reached out to him privately, as he disabled public comments. His Instagram posts related to the coronavirus had thousands of likes, and his profile gained more than ten thousand followers in March 2020. In an interview with *Día D*, he explains that he had to close his Instagram account due to the influx of hate comments. The negative comments affected his well-being and mental health, but he still saw it as his responsibility to provide information and guidance to the public. He tried to empathize with the audience and referred to his “self-esteem” as the reason he overcame the criticism. He also recognized that the negative attention would have affected others more severely (Día D, 12 Apr. 2020).

The journalists and patient zero acknowledged the term “patient zero” presented limitations, yet they continued to use the term in their communication. The Peruvian patient zero experienced a contradictory scenario, where he recognized himself as patient zero, assuming a self-imposed responsibility while acknowledging potential previous cases. In the April 12 interview, the patient and his family acknowledged the possibility that he was not the index case, as there may have been multiple introductions of the virus before his diagnosis. The interview also featured an epidemiologist’s testimony stating that “he is the patient zero that was captured by personal choice,” meaning that it was highly likely that he was not the index case. The interview ends with a final commentary from the program’s host, who said that “patient zero stood in the spotlight to give the warning signal that we were facing a virus still under study and with no vaccine in sight” (Día D, 12 Apr. 2020).

The Peruvian patient zero’s social media publications and interviews influenced the public’s perception of him, changing from negative to positive. The public was interested in his testimony because he provided the patient’s perspective, something only few could do at the time. His personal experience gave him credibility and provided information that complemented expert knowledge. Comparing the commentary section from his two interviews with *Día D*, we can see a change in the public’s response. In the first anonymous interview, users described him as irresponsible. In the second interview, most comments thanked him for following the doctor’s indications and revealing his identity so people could learn about the virus. A user said, “Luis Felipe was not the first case in Peru, a lot of people must have been infected, but he was responsible” (Rovegno, 13 Apr. 2020). Other users expressed how his testimony made them feel hopeful after seeing “this young man and his family are now healthy” (Quijandria, 13 Apr. 2020).

Multiple factors can explain why the public response to patient zero’s testimony changed when he revealed his identity and more information about the virus became known. One factor is that the patient overcame the coronavirus after following medical and government mandates, and authorities described him as a model patient. People also reacted positively to his testimony since social media users bullied him after exposing his identity to offer information about the coronavirus. Another possible answer is that the authorities identified other patients with no relationship to him after his diagnosis, showing multiple entrances of the virus. When patient zero appeared on television, the government had already implemented measures to control the virus. This might have indicated to the audience that the responsibility was not exclusively his.

When patient zero used his social media, he shared his experience and influenced an audience with positive and adverse reactions. People were interested in his experiences but contacted him also for medical advice to overcome the infection, knowing he was not a doctor. In the case of the Peruvian patient zero, his posts aligned with the scientific evidence of the time and with the government mandates of isolation and sanitary protocols. However, he could have used his channel to promote pseudoscientific treatments, spread misinformation, or provide medical advice. Although the government and the media created an interest in the patient zero, they could not intervene on his social media to fact-check his information since he was not an institutional channel. When the authorities support the figure of a patient zero, they create an interest in a person who might question them or experts. This creates a potential conflict for audiences looking for information on the coronavirus.

The public’s interest in patients zero provides the public with an alternate view of the pandemic that authorities and experts should not overlook. The patients might support scientific narratives or question them. Public health officials should be aware of the patient zeros’ role as actors in the pandemic, preventing conflicts and supporting them. The Peruvian patient zero had media support since his first appearances were in a program hosted by a family friend who first respected his privacy but also might have offered him some guidance. The Peruvian patient zero recognized the negative aspects of revealing his identity and how anyone in the same position would have experienced a severe impact on their mental health. Public health officials should include guidance for patients zeros to overcome the pressure put on these patients, even when experts stress that they might not be the first patients and that their fame might be momentary.

The Peruvian patient zero’s media appearances and social media posts illustrate how the concept of patient zero responded to social interests rather than scientific ones. The consequence of using this term affects the patients and the efforts to control the pandemic. The narratives about patients’ involvement and their socioeconomic situation shaped how the public interpreted the disease at the beginning of the pandemic. In the case of Peru, the first patient’s wealth, age, occupation, and commitment to following the rules were disclosed to the public, which shaped the public’s image of the patient zero. Even when the audience first criticized him, they still found his testimony valuable to understand the covid-19 disease. The media interviews and patient zero’s Instagram posts helped build the narrative of a heroic and responsible citizen who helped others with his testimony rather than staying anonymous. In the case of the Peruvian patient zero, he responded to the public’s interest by providing more information to the media and opening his social media account for the audience to interact with him.

## Final considerations

The advancement of the coronavirus during the first weeks of 2020 moved governments to identify and announce the first coronavirus cases in each country. The term patient zero gained notoriety as a synonym for the beginning of the pandemic in each country. Media and authorities used the term interchangeably to indicate the first identified case, the first case ever in a country, and the first case worldwide. As scientists indicate, the term does not respond to scientific interests because it equates the index case with the primary case. Despite its inaccuracy, the term patient zero was used in several countries to signal the beginning of the pandemic.

In the case of Peru, the patient zero became a relevant actor for the early months of the pandemic. His case shows how patients identified as the first case were subjects of interest from the public and authorities since their experience became relevant for the public to understand the pandemic. The Peruvian patient zero was considered a valid source of knowledge since his experience with the coronavirus resulted from his personal experience rather than scientific knowledge. The government amplified his case, using it to predict how other people would react to the virus when they described him as a model patient.

The Peruvian patient zero used traditional and social media to establish his agency by reinforcing his image as a responsible citizen. He participated in interviews, first anonymously and later as a public figure, and established direct communication with the audience using Instagram to share his symptoms, medical treatment, and family details. People could message him directly through Instagram, asking questions or criticizing him. In his second interview, he mentioned being aware of the term’s vagueness; however, he continued to use it and allowed others to identify him as such. He saw a greater good in providing information to the audience rather than staying anonymous, even when the public strongly criticized him.

Creating public interest in the patient zero ignores the negativity these patients might receive and overlooks the responsibility of involving non-expert actors as central characters of the pandemic. Audiences can be critical of patients zero, affecting their well-being and judging them based on their medical outcomes. Moreover, patients zero can become problematic if they use their new status as a person of interest for economic profit, to promote pseudoscientific methods, or spread misinformation.

The Peruvian patient zero shows how involved patients are in their medical conditions and how they become public figures to establish their agency. The audience’s response to them goes beyond listening to their testimony. People engage with patients zero by reaching out to them or expressing their opinions on social media posts. The social media comments, the media coverage, and the authorities’ use of a patient zero as a model patient reinforced the importance of these patients as crucial actors during the covid-19 pandemic. Even when imprecisely identified as the first patient, they still shaped the audience’s interpretation of the early days of the pandemic, for which public health officials should not overlook their influence.
